# Effect of Caffeine on the Repeated Modified Agility Test from Some Cardiovascular Factors, Blood Glucose and Rating of Perceived Exertion in Young People

**Published:** 2017-06

**Authors:** Nidhal JEBABLI, Nejmeddine OUERGHI, Jihen BOUABID, Ramzi BETTAIB

**Affiliations:** 1. Research Unit, Sportive Performance and Physical Rehabilitation (S2PR), High Institute of Sports and Physical Education, University of Jendouba, Kef, Tunisia; 2. Dept. of Life Sciences, Faculty of Sciences of Bizerte, Zarzouna Bizerte, Tunisia; 3. Higher Institute of Applied Studies in Humanity, University of Jendouba, El-kef, Tunisia

**Keywords:** Caffeine, Performance, Cardiovascular factors, Blood glucose, RPE

## Abstract

**Background:**

The aim of this study was to compare the effects of the ingestion of either the caffeine (CAF) or the placebo (PLA) on performance of repeated modified agility test (RMAT), some cardiovascular factors, metabolic and notes of perceived exertion (RPE) in young males and females.

**Methods:**

In a randomized double-blind study, we enrolled 18 active students (10 males and 8 females) in Sport Sciences pursuing degrees in Exercise Science and Physical Education at the University of Sports of Kef (Tunisia), during the academic year 2013–2014. All participants were ingested CAF (5 mg.kg^−1^) or PLA 60 min before performing an RMAT. Total times (TT), peak time (PT) and fatigue index (FI) were identified as the RMAT indices. Heart rate (HR), arterial pressures (PA), blood glucose (BG) and RPE were assessed before, during and after the RMAT.

**Results:**

Taking caffeine had been improved the performance by the significant decreased of TT on male gender better than female gender and the entire group. In addition, there was a significant improvement on HR during and after RMAT in both genders and the whole group, except after RMAT among male gender. However, the repeated measurement results had demonstrated no effect of caffeine on PA, BG and RPE.

**Conclusion:**

Caffeine supplement had a beneficial effect on agility performance and HR in male better than in female, although, there was no improvement in PA, BG and RPE.

## Introduction

Since 2004, caffeine (1, 3, 7-Trimethylxanthine) had been used as an ergogenic substance, not doping, legally by the World Anti-Doping Agency (WADA). Caffeine (CAF) was effective in improving the performance of various types including aerobic exercises (1–3), activity of high-intensity sports team (4, 5) and strength (6, 7). The ideal dose of CAF that could affect physical and athletic performance is 5 mg.kg^−1^ (8).

In the context of high-intensity intermittent exercise, the results were equivocal. Indeed, some authors had shown a beneficial effect of CAF on performance (9–11). Others had demonstrated no significant effect in the CAF conditions (12, 13). In the same context, scientific researches suggested that caffeine could influence the cardiovascular (14, 15), metabolic parameters (16, 17) and perceived exertion (RPE) (18, 19). The effect of CAF on certain factors cardiovascular [heart rate (HR), systolic blood pressure (SBP), diastolic blood pressure (DBP), mean arterial pressure (MAP)], blood glucose (BG) and the perceived exertion (RPE), for each type, had not been specifically studied before and after high-intensity intermittent exercise.

The aim of this study was to determine the effect of the ingestion of CAF on the performance of repeated modified agility test (RMAT) (20) from certain cardiovascular factors, blood glucose and RPE in male and female active participants.

## Materials and Methods

### Study subjects

In this study, we enrolled 10 healthy male and eight female participants, in Sport Sciences pursuing degrees in Exercise Science and Physical Education at the University of Sports of Kef (Tunisia), during the academic year 2013–2014. All participants were volunteers and were briefed on the stages of our study, approved by the Scientific Committee of the Higher Institute of Sport and Physical Education of Kef. The protocol was carried out in accordance with the Declaration of Helsinki. Table 1 shows the descriptive data of participants.

**Table 1:** Descriptive data of participants

	**Age (yr)**	**Weight (kg)**	**Height (m)**	**BMI (kg.m^−2^)**
Male (n=10)	22.9 ± 1.46	66.2 ± 5.89	1.76 ± 0.05	21.4 ± 1.98
Female (n=08)	21.8 ± 0.45	60.7 ± 3.77	1.64 ± 0.03	22.4 ± 1.54

Data are expressed as means ± SD; BMI, body mass index

### Experimental protocol

**Fig. 1:**
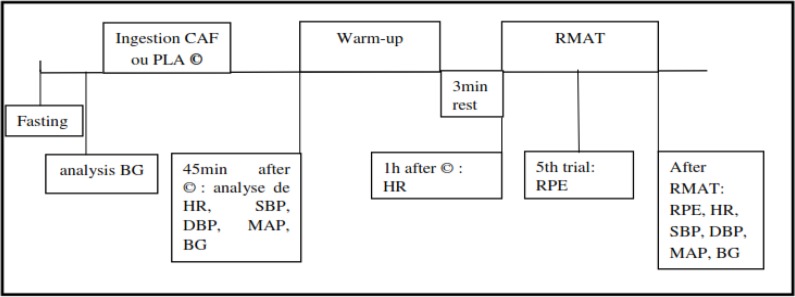
The time course of experimental protocol CAF: caffeine; PLA: placebo; HR: heart rate; SBP: systolic blood pressure; DBP: diastolic blood pressure; MAP: mean arterial pressure; BG: blood glucose; RPE: notes of perceived exertion; RMAT: repeated modified agility test

In this double blind, randomized study, all participants performed two experimental sessions under different conditions: (i) placebo (PLA) or (ii) CAF. These experimental sessions were conducted during two consecutive weeks, the same day and at the same time for each subject. Each experimental session began with an analysis of fasting glucose. Then taking 5 mg.kg^−1^ CAF or PLA. After 45 min of ingestion (CAF or PLA), the participants warmed up 10 min. Next, they performed RMAT test after a 2–3 min recovery. Total time (TT), peak time (PT) and fatigue index (FI) was identified as the RMAT indices. Furthermore, HR was measured during RMAT using a heart rate monitor (Polar TF4). Moreover, the rating of perceived exertion (RPE) was recorded in the 5^th^ and the 10^th^ repetition of the RMAT by using the Borg scale (1998). SBP and DBP were measured before and after the RMAT using a pressure monitor (GTEST Diagnostic Bras KD 591). Mean arterial pressure (MAP) was then calculated using the following formula: MAP =PAD + 1/3 * (PAS - PAD). The analysis of blood glucose was evaluated before and after the RMAT by using the colorimetric enzymatic method on an auto-analyzer Architect C8000 (Abbott Laboratories. Abbott Park. IL. USA). Fig. 1 represents the time course of experimental protocol. Verbal encouragements were given to subjects during the experimental process.

### Repeated modified agility test (RMAT)

**Fig. 2:**
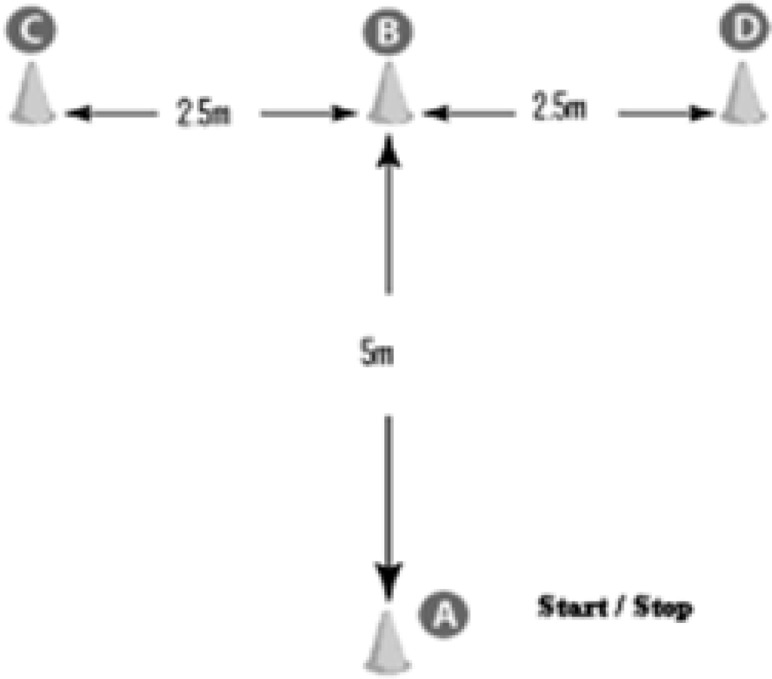
Repeated modified agility test

The RMAT consisted of 10 maximal sprints of 20 m with 25 sec rest period (Fig. 2). Each sprint involved 4 changes of direction with 3 displacement modes: forward, lateral and backward (20). The RMAT performances were recorded using an electronic timing system (Globus, Microgate).

### Statistical procedures

After normality and homogeneity assurance, the differences between the means had been evaluated by using two-ways of analysis of variance for repeated measurements. If this analysis showed significant differences, the means would be comparing post hoc using Bonferroni correction. The effect size (d) was calculated using Cohen’s d (≤0.2. small. 0.5 to 0.79. moderate; ≥0.8. large). The paired samples *t*-test had been performed on the HR and the performance indices. Besides, significance was set at *P*<0.05* and *P*<0.01**. Thus, all values were expressed as means ± standard error of the mean (means ±SD). In addition, statistical analyses were performed using Statistical Package for the Social Sciences ver. 16.0 software (SPSS Inc., Chicago. IL).

## Results

### Performance

No significant difference had been observed for TT, PT, FI between PLA and CAF for both male and female genders except the TT in the male gender in the caffeine condition. A small improvement was observed for all TT indices PT and FI for the male gender (5.49%, 5.16%, −0.22%, respectively) and female gender (2.95%, 1.02%, −2.08%, respectively) with weak effects sizes (Table 2).

**Table 2:** The effect of caffeine or placebo on RMAT performance indices

	**Male**			**Female**			**Total**		
	**Caffeine**	**Placebo**	**P**	**Caffeine**	**Placebo**	**P**	**Caffeine**	**Placebo**	**P**
TT(s)	64.3±4.14	68.1±6.88^*^	0.028	71±2.14	73.3±3.51	0.08	67.1±4.79	70.2±6.14	0.006
PT(s)	6.1±0.30	6.4±0.6	0.091	6.8±0.27	6.9±0.37	0.345	6.4±0.46	6.6±0.56	0.007
FI (%)	6.2±3.29	6.4±2.64	0.735	4.4±2.08	6.5±4.69	0.345	5.4±2.88	6.4±3.43	0.388

Data are expressed as means ± SD; TT: Total time; PT: peak time; FI fatigue index; RMAT: repeated modified agility test.

* *P*<0.05 the difference is significant between the caffeine and placebo

### Heart rate

Before RMAT, no significant difference had been observed between CAF and PLA provided for both genders and the whole group. For the HR mean, a significant difference was noticed between the condition PLA and CAF for the female gender (*P*=0.043, d=0.118) and the whole group (*P*=0.005, d=0.002), whereas there was no difference observed between CAF and PLA in the male group.

During the RMAT, there was a significant difference for the HR maximum, between the two conditions for both genders, male, female, and the whole group (*P*=0.027, d=0.190; *P*=.042, d=0.071 and *P*=0.003, d=0.001, respectively). After RMAT, a significant difference was observed between CAF and PLA conditions for female gender (*P*=0.043, d=0.214) and the whole group (*P*=0.008, d=0.003), whereas no significance had been observed for the male gender. No significant difference had been revealed between the two genders before, during and after the RMAT (Table 3).

**Table 3:** The effect of caffeine or placebo on some cardiovascular factors, blood glucose, and notes of perceived exertion before, during and after the RMAT

**Variable**	**Male**			**Female**			**Total**		
	**Caffeine**	**Placebo**	***P***	**Caffeine**	**Placebo**	***P***	**Caffeine**	**Placebo**	***P***
**HR (Beat/min)**									
Pre-test	114.1±27.36	113±29.81	1	114.4±24.88	112.8±6.3	0.893	112.9±22.34	114.3±25.17	0.875
Maximum	169±1.06	157.1±12.35	0.027*	170.2±9.91	165±11.25	0.042*	160.4±12.06	169.5±11.99	0.003**
Mean	146.9±16.43	138.8±16.33	0.630	153±14.47	144.2±12.01	0.043	141.1±14.33	149.4±15.26	0.005
Post-test	155.1±15.45	145.3±18.45	0.068	168.8±10.28	156.8±14.97	0.043*	150.1±17.39	160.8±14.77	0.008**
**SBP (mmHg)**									
Pre-test	116.4±15.41	123.3±11.29	0.428	116.6±9.91	127.8±11.97	0.406	120.5±10.83	121.2±14.70	0.807
Post-test	133.1±6.31	131.4±15.55		131.6±7.92	128.2±15.99		132.5±6.72	130.1±15.09	
**DBP (mmHg)**									
Pre-test	73.1±18.32	77±10.54	0.848	75.4±5.22	77.8±3.11	0.686	74.4±13.92	77.3±8.02	0.975
Post-test	79.7±8.36	77.3±12.70		80.6±4.39	77.2±6.26		80.1±6.73	77.3±10.11	
**MAP (mmHg)**									
Pre-test	90.2±12.85	90.1±11.87	0.405	89.13±6.48	94.4±4.98	0.569	89.8±10.28	91.9±9.53	0.806
Post-test	97.5±6.48	95.3±11.67		97.5±4.88	94.2±5.06		97.5±5.62	94.9±9.16	
**BG (mg/dl)**									
Fasting	84±3.96	84.1±4.14		82.6±4.72	83±4.53		83.4±4.14	83.7±4.14	
Pre-test	95.7±8.28	98.7±14.37	0.850	92.6±5.03	94.8±8.98	0.121	90.3±9.60	97.1±12.09	0.288
Post-test	100.3±10.50	102±24.75		100.6±10.11	103±12.59		101.4±10.94	101.4±19.28	
**RPE**									
5th trial	10.3±1.50	10±2.89	0.407	11.4±0.89	12.2±3.35	0.749	10.8±1.36	10.9±3.15	0.658
10th trial	11.4±1.62	12±1.73		13.2±1.1	13.6±2.41		12.2±1.64	12.7±2.10	

Data are expressed as means ± SD; HR: heart rate; SBP: systolic blood pressure; DBP: diastolic blood pressure; MAP: mean arterial pressure; BG: Blood Glucose; RPE: notes of perceived exertion; RMAT: repeated modified agility test.

* *P*<0.05 significant difference between caffeine and placebo;

** *P*<0.01 Very significant difference between caffeine and placebo

### Arterial pressure

Before and after the RMAT, there was no significant difference found between the two PLA and CAF for both genders. Even when using repeated measures three-ways (supplement X Gender X period) analysis of variance, there was no significant difference had been observed between the two conditions PLA and CAF (Table 3).

### Blood glucose

By using a repeated measures three-way (supplement X Gender X period) analysis of variance, no significant difference (*P*=0.778, d=0.095) had been observed (Table 3).

### The perceived exertion

No significant difference was detected by repeated measures three-way (supplement X Gender X period) analysis of variance (Table 3).

## Discussion

This study aimed to investigate the effect of gender and consumption of CAF compared with PLA on the performance of RMAT from certain cardiovascular factors, BG and RPE.

The results showed three main things. First, there was a unique index had been improved for the male gender (TT). Second, there was improved significantly on HR maximum in male gender, as well as on HR mean, HR maximum and HR post-test for the female gender and the whole group. Finally, there was no significant effect of consumption of CAF or PLA on arterial pressure, blood glucose and the RPE for both male and female genders.

Caffeine was widely used in sports compound. It had an ergogenic effect on the performance of high-intensity intermittent exercise. Several studies demonstrated the effectiveness of CAF on agility performance (9–12). In addition, beneficial effect of CAF was shown in a significant increase in TT, PT and FI for a period of 5 sets of 6 * 20 m (25 or 60 sec recovery; 5 min rest) in active men (11). All of these results were consistent with our results in the index TT for the male gender. This significant performance increase (*P*<0.05) could be related mainly to the improvement of neuromuscular activity by improving neural transmission (1). CAF had been stimulated the central nervous system with increased vigilance, led to more concentration during the execution of RMAT specifically when changing directions (21). However, our results of PT and IF index were consistent with other studies (12, 13) revealed no significant effect (*P*>0.05) of CAF on performance in repeated sprint ability. No study was interested in the unique effect of CAF on repeated sprint performance in the female gender. However, Red Bull contains 80-mg portion of CAF, 27-g carbohydrate and 1-g taurine that did not change the performance of repeated sprints but had minimal effect in the female gender (22). The minimal effect (not significant) of CAF ingestion on repeated sprint performance was revealed in several studies (12, 13, 22) that were not compatible with the significant improvement in our results on both female and male genders. The controversies between these studies may be due to either the sex selection or the selected study population (athletic, active and sedentary).

The present study demonstrated a significant improvement on HR mean, HR maximum and HR post-test in female gender and the whole group. However, only in the male gender HR mean was significantly improved. These results were similar to another study (15) who demonstrated that CAF increased on HR during exercise. The increase seemed to be linked mainly to the inhibition of phosphodiesterase and the accumulation of cyclic adenosine monophosphate (23); as a result, there was an improvement in the myocardial contractility and improved HR. However, our results were in disagreement with another study (14). They suggested that CAF might affect only HR and blood pressure during the passive recovery, but not during the exercise. This disagreement had been observed for the female gender by other studies too (22, 24–26), which had shown no significant change in HR during exercise in the CAF condition.

The CAF promoted an almost identical improvement in SBP and DBP on women and men, without significant difference on SBP vice versa for PAD in male compared to female gender (27). All our results showed some disagreements with this study by the non-significant increase in SBP, DBP and MAP by gender.

In addition, few studies had assessed the effect of CAF on blood glucose. Indeed, a recent study (17) did not indicate any significant changes in blood glucose before and after muscular exercise for CAF condition. All these results were consistent with our results that revealed no significant difference between CAF and PLA requirements for both female and male genders. Our study was also confirmed by other scientific studies that demonstrated that the CAF had no relationship between the increase of the adrenaline and the blood glucose (14, 27, 28). For the perceived exertion (RPE), our results were similar to several studies that demonstrated no significant effect of CAF on the RPE of the male gender (15, 19) and for the female gender (16, 29).

The present study had a number of limitations. For example, the relatively small number of participants may have been underpowered by the study.

## Conclusion

The caffeine supplement seemed to have beneficial effect on the performance agility on male than on female. This supplement had no effect on blood pressure and glucose, as well as on RPE in both genders, unlike on HR during RMAT. Besides, caffeine promote improved an agility of performance on the male better than the female gender.

## Ethical considerations

Ethical issues (Including plagiarism, informed consent, misconduct, data fabrication and/or falsification, double publication and/or submission, redundancy, etc.) had been completely observed by the authors.
